# An Electrochemical Immunosensor Based on a Self-Assembled Monolayer Modified Electrode for Label-Free Detection of α-Synuclein

**DOI:** 10.3390/s20030617

**Published:** 2020-01-22

**Authors:** Chuang-Ye Ge, Md. Mahbubur Rahman, Wei Zhang, Nasrin Siraj Lopa, Lei Jin, Sujin Yoon, Hohyoun Jang, Guang-Ri Xu, Whangi Kim

**Affiliations:** 1 Department of Energy and Materials, Konkuk University, Chungju 380-701, Korea; jiyuanqiaohe2010@hotmail.com (C.-Y.G.); mahbub1982@kku.ac.kr (M.M.R.); arno_zw@hotmail.com (W.Z.); lopa_2988@yahoo.com (N.S.L.); jinlei8761@naver.com (L.J.); ysj920126@naver.com (S.Y.); 200417450@kku.ac.kr (H.J.); 2 Department of Chemistry and Chemical Engineering, Henan Institute of Science and Technology, Xinxiang 453003, China; xugr70@163.com; 3 Department of Liberal Arts, Konkuk University, Chungju-si 27478, Korea; 200417450@kku.ac.kr

**Keywords:** α-synuclein, cystamine, immunosensor, self-assembled monolayer, fluorine-doped tin oxide

## Abstract

This research demonstrated the development of a simple, cost-effective, and label-free immunosensor for the detection of α-synuclein (α-Syn) based on a cystamine (CYS) self-assembled monolayer (SAM) decorated fluorine-doped tin oxide (FTO) electrode. CYS-SAM was formed onto the FTO electrode by the adsorption of CYS molecules through the head sulfur groups. The free amine (–NH_2_) groups at the tail of the CYS-SAM enabled the immobilization of anti-α-Syn-antibody, which concurrently allowed the formation of immunocomplex by covalent bonding with α-Syn-antigen. The variation of the concentrations of the attached α-Syn at the immunosensor probe induced the alternation of the current and the charge transfer resistance (*R_ct_*) for the redox response of [Fe(CN)_6_]^3−/4−^, which displayed a linear dynamic range from 10 to 1000 ng/mL with a low detection limit (*S*/*N* = *3*) of ca. 3.62 and 1.13 ng/mL in differential pulse voltammetry (DPV) and electrochemical impedance spectra (EIS) measurements, respectively. The immunosensor displayed good reproducibility, anti-interference ability, and good recoveries of α-Syn detection in diluted human serum samples. The proposed immunosensor is a promising platform to detect α-Syn for the early diagnose of Parkinson’s disease, which can be extended for the determination of other biologically important biomarkers.

## 1. Introduction

According to a projection of the World Health Organization, neurological disorders and diseases are one of the principal causes of disability and death worldwide, including Parkinson’s, Alzheimer’s, Creutzfeldt-Jakob, Huntington’s, and Wilson’s diseases [1,2]. In particular, Parkinson’s disease (PD) is the second leading cause of the neurodegenerative disorder, which affects about 1 and 4% of the people above the ages of 60 and 80 years old, respectively [3]. Nowadays, people at the age of below 50 years old are also affected with PD [4]. PD is a chronic and incurable neurodegenerative disorder with the progressive loss of neuronal cells in the substantia nigra [5,6]. The population with PD is anticipated to rise between 8.7 to 9.3 million by 2030 [7]. Thus, early diagnosis and adequate tracking of PD to retard its progression are substantially important, which concurrently improves the quality of a PD affected people’s life by reducing the overall treatment cost [8].

Several widely tested biomarkers have been investigated intensively for several decades to diagnose PD with high accuracy, including α-synuclein (α-syn), protein deglycase (DJ-1), Leucine-rich repeat kinase 2 (LRRK2), amyloid-beta, apolipoprotein A1 (ApoA1), dopamine (DA), and glutathione [9,10,11]. In particular, α-Syn, is a cytoplasmic protein with 140-amino acids, implicating the pathogenesis of PD. This is because α-Syn can easily aggregate and form insoluble fibrils which are the principal components of Lewy body and Lewy neuritis at the pathological conditions [12]. α-Syn can be predominantly expressed in the human brain in the thalamus and cerebellum, hippocampus, and neocortex. Recent studies reveal that α-Syn can be secreted toward the extracellular matrix of the human brain, which induces the spreading of the pathology of PD to the extracellular matrix of the human brain by propagating in a prion-like mechanism [13]. Concurrently, the concentration of α-Syn can be increased in the body fluids of PD patients, including cerebrospinal fluid (CSF) and blood plasma. Motivated by the existence of an elevated level of α-Syn concentration in the blood plasma of a PD patient, researchers have been encouraged for the direct detection of α-Syn concentration in blood plasma for the early diagnosis of PD. Consequently, different analytical techniques have been demonstrated for the detection of α-Syn, including mass spectrometry [14], nuclear magnetic resonance spectroscopy [15], fluorescence [16], and capillary electrophoresis [17], photoelectrochemical [18], surface plasmon resonance [19], and electrochemical methods [19,20,21,22,23]. 

Among these methods, electrochemical detection methods are highly advantageous due to their high sensitivity, low-cost, easiness for miniaturization, and point-of-care testing. Moreover, the electrochemical methods show great potential for in-vivo and in-vitro detection [24,25,26]. With the rapid advancements of bio-electrochemical technology and nanomaterials, electrochemical immunoassay based on the natural recognition between antigen and antibody appears to be the most promising method [27,28]. To the best of author’s knowledge, few reports are available for the electrochemical immunoassay based detection of α-Syn. For example, Karaboğa demonstrated an AuNP-polyglutamic acid (PGA)-modified indium-doped tin oxide electrode (ITO) for the immobilization and binding of anti-α-Syn-antibody and α-Syn antigen, respectively [22]. This label-free PGA/AuNP/ITO based nanoimmunosensor is promising for the disposable type detection of α-Syn antigen with a low detection limit. In another report, An et al. developed a polyamidoamine dendrimer-encapsulated Au nanoparticle (PAMAM-AuNPs) bound poly-o-aminobenzoic acid (poly-o-ABA)-modified glassy carbon electrode (GCE) [23]. The existence of free amine (–NH_2_) groups onto the PAMAM surface allow the covalent attachment of α-Syn antigen. Subsequently, horseradish peroxidase-secondary antibody functionalized AuNPs labels were immobilized onto the sensor surface, which produced an electrocatalytic signal by the reduction of H_2_O_2_ in the presence of horseradish peroxidase mediated oxidized thionine. Even though this label-based electrochemical immunoassay showed high sensitivity and stability for the detection of α-Syn, nevertheless, the complex fabrication and high materials cost of this method will hinder the practical application of this immunosensor for α-Syn detection. Therefore, it is indispensable to develop a label-free and low-cost electrochemical immunoassay platform by the reduction of the fabrication steps and materials cost for the future commercialization of the α-Syn detection system. 

**Figure 1:**
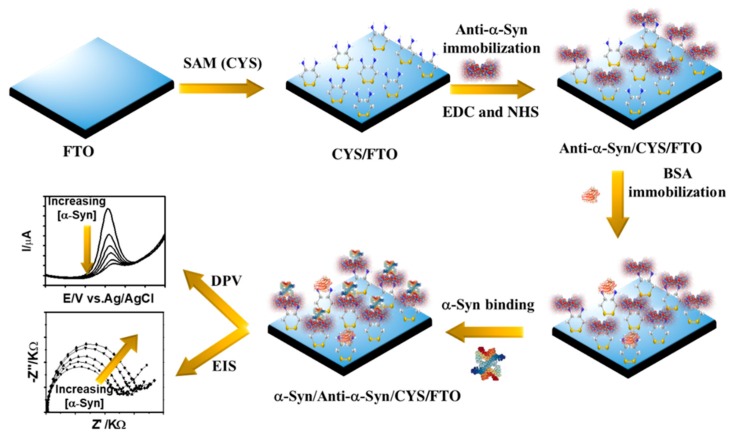
Schematic representation of the α-synuclein (α-Syn) immunosensor.

Herein, we fabricated a simple, low-cost, and label-free immunosensor for the detection of α-Syn based on a self-assembled monolayer (SAM) modified fluorine-doped tin oxide (FTO) electrode. The schematic representation of the fabrication of the α-Syn immunosensor together with the detection systems is shown in Figure 1. The molecular-level interaction between the CYS and FTO during SAM formation allows the adsorption of the CYS with a high amount. The CYS-SAM modified FTO surface enables to immobilizing anti-α-Syn antibody by the amide bonds between the –NH_2_ groups of CYS and carboxyl groups (–COOH) of anti-α-Syn, which induces the formation of immunocomplex between anti-α-Syn antibody and α-Syn-antigen. Concurrently, the immunosensor exhibits high sensitivity for the detection of α-Syn antigen, which was detected by monitoring the change of current and the charge transfer resistance (*R_ct_*) for the redox reaction of [Fe(CN)_6_]^3−/4−^.

## 2. Materials and Methods

### 2.1. Chemicals and Reagents

CYS, recombinant α-Syn human, monoclonal anti-α-Syn antibody from mouse, immunoglobin G (IgG) from human serum, cholesterol (Chol.), glucose oxidase (GOx), bovine serum albumin (BSA), DA, ascorbic acid (AA), uric acid (UA), 1-ethyl-3-3(3-dimethylaminopropyl) carbodiimide (EDC) disodium hydrogen phosphate (Na_2_HPO_4_), sodium dihydrogen phosphate (NaH_2_PO_4_), and N-hydroxysuccinimide (NHS) were purchased from Sigma-Aldrich (St. Louis, MO, USA). The phosphate buffer (PB, 0.1 M) saline with pH = 7.0 was prepared by mixing an appropriate amount of Na_2_HPO_4_ and NaH_2_PO_4_, according to a previously reported method [24]. All of the solutions used in this experiment were prepared using ultrapure water, which was acquired from a water purifying system (18 MΩ·cm). 

### 2.2. Instrumentation and Measurements

A potentiostat (Ivium-n-Stat, Ivium Technologies, The Netherlands) was used to conduct all the electrochemical measurements. FTO substrate (8 Ω/Sq. TEC8, Pilkington, Tokyo, Japan), a platinum wire, and an Ag/AgCl electrode were used as working, counter, and the reference electrode, respectively. Differential pulse voltammetry (DPV) was measured with the pulse height, pulse width, and pulse period of 100 mV/s, 2 ms, and 100 ms, respectively. The Nyquist plot form of electrochemical impedance spectra (EIS) was measured by an impedance analyzer (Zahner-Elektrik GmbH & Co., Kronach, Germany) in the frequency range of 1 MHz–0.1 Hz with the sinusoidal wave amplitude of 5 mV. Simulation of Nyquist plots with the Randles circuit model was executed using Z-view software (Scribner Associates Inc., Southern Pines, NC, USA). All the electrochemical experiments were performed in PB solution with pH = 7.0 at 25 °C unless noted otherwise. The wetting properties of the electrodes were characterized by a contact angle measurement system (CAM, SEO-300A, s-eo, Suwon-si, Korea). The surface topography and roughness of the FTO and CYS/FTO electrodes were analyzed by an atomic force microscope (AFM) (Nanoscope-IV, Digital Instruments, Tonawanda, NY, USA). 

### 2.3. Preparation of Anti-α-Syn/CYS/FTO Probe

Before the formation of CYS-SAM, a piece of FTO substrate (2 cm × 1.5 cm) was cleaned by ultrasonication in a dilute aqueous solution of Triton X-100 for 30 min. Then, the FTO electrode was rinsed by acetone and ethanol consecutively, and dried by N_2_ purging. For the preparation of CYS-SAM, the cleaned FTO substrate was placed into a solution of CYS (50 mM) in ethanol for 4 h, washed with PB solution, and dried by N_2_ gas. During the course of experiments, the geometric surface area of all electrodes was confined to ca. 0.32 cm^2^ by an O-ring (with negligible distortion or deformation) in an electrochemical cell and used for SAM formation and the binding of antibody and antigen [24]. The unoccupied surface of FTO electrode was secured due to the complete isolation of the analyte solution which did not show any interference to the performance of the immunosensor. The CYS/FTO electrode was incubated into an anti-α-Syn (1 µg/mL) antibody solution in PB at 4 °C for 12 h together with EDC/NHS (20 mg/mL and 15 mg/mL, respectively) activators [29]. The anti-α-Syn/CYS/FTO electrode was washed with PB and further immersed in a solution of BSA (0.1% in PB) for 5 h to prevent the non-specific binding of the target α-Syn antigen. The anti-α-Syn/CYS/FTO immunosensor was incubated into α-Syn antigen solution in PB with varying concentrations at 4 °C for 50 min [22]. The unbound α-Syn antigen was removed from the surface of the immunosensor by washing with PB solution, which was directly used for electrochemical measurements. 

## 3. Results and Discussion

### 3.1. Contact-Angel and AFM Characterizations of CYS-SAM

**Figure 2:**
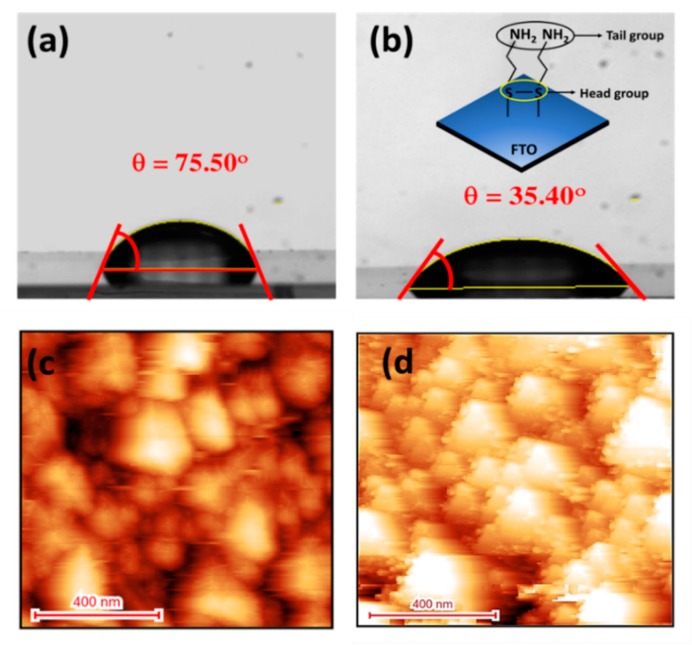
(**a**,**b**) Contact angle images of water droplets for bare fluorine-doped tin oxide (FTO) and cystamine (CYS)/FTO, respectively (insets of b shows the interaction pathways of CYS with the FTO surface). Non-contact mode AFM images of (**c**) bare FTO and (**d**) CYS/FTO electrodes.

The formation of SAM of a thiol compound onto tin-oxide (SnO_2_) is occurred by the adsorption of thiol molecules. However, the surface coverage of alkane-thiol due to the SAM formation onto SnO_2_ is much less than the FTO surface [30]. This suggests that fluorine dopant in the FTO plays a crucial role in assembling thiolated compounds. To elucidate the successful CYS-SAM formation onto the FTO electrode, we measured the contact angles (CA) of the electrodes using water, as shown in Figure 2a,b. Un-doped SnO_2_ usually exhibits super hydrophilic behavior with extremely low CA (θ = 20°) [31], while bare FTO electrode displays much improved hydrophobic property with the CA value of ca. 75.50°. This can be attributed to the existence of hydrophobic fluorine dopants in the FTO. The CA of the CYS/FTO electrode was decreased significantly to ca. 35.40°, which demonstrated the hydrophilic nature of the SAM modified surface. This can be assigned to the H-bonding interaction of water with the tail –NH_2_ group of CYS-SAM molecules. Considering a very small difference in the electronegativity between S of CYS and H in water (ca. 0.4), it is more appropriate to consider the hydrogen bonding interaction between –NH_2_ group of CYS and H_2_O that decreased the CA. This designates that the CYS-SAM was formed by the interaction of tail sulfur with the SnO_2_ and fluorine of FTO, which is well-agreed with the previous studies [30,31].

The surface topography of the bare FTO and CYS/FTO electrodes was studied by non-contact AFM, as shown in Figure 2c,d. Bare FTO electrode displays the presence of close-packed F-doped SnO_2_ particles. For the bare FTO, the average surface roughness was ca. 27.50 nm, which is consistent with the reported value [32]. The particle size of the FTO was in the range from ten to several hundred nanometers, which is advantageous to obtain a high density of CYS-SAM onto the FTO electrode. Upon the formation of CYS-SAM, the surface roughness of the electrode was increased to ca. 29.80 nm. Additionally, the AFM image also clearly shows the existence of CYS molecules layers onto the F-doped SnO_2_ particles. These results specify the successful CYS-SAM monolayer formation onto the FTO surface.

### 3.2. Electrochemical Characterization

**Figure 3:**
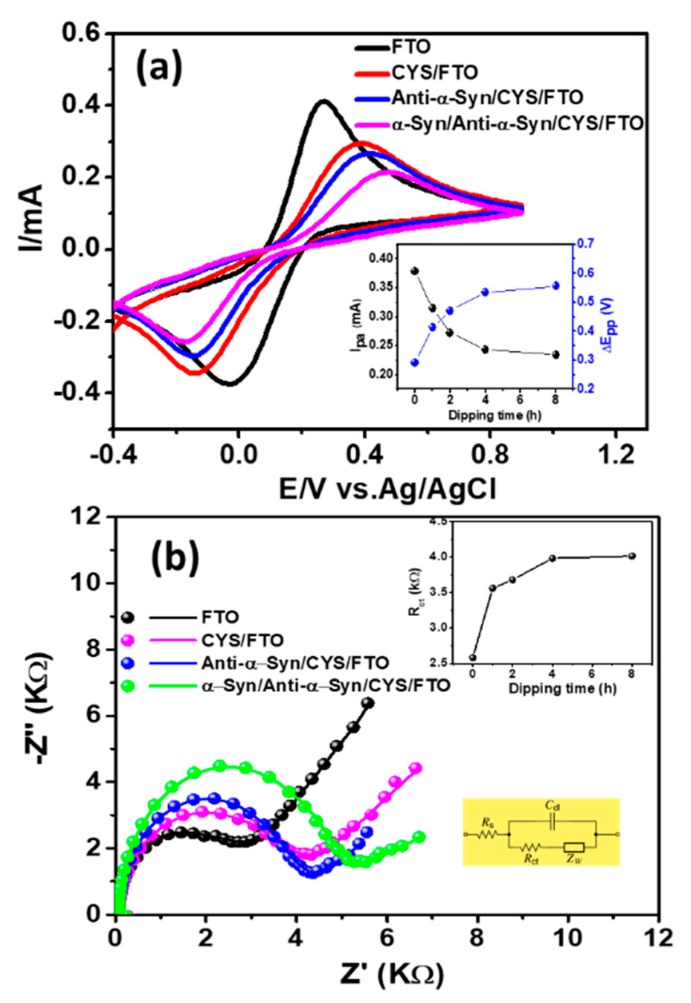
(**a**) Cyclic voltammograms (CVs) (scan rate 100 mV/s) and (**b**) electrochemical impedance spectra (EIS) plots of FTO, CYS/FTO, anti-α-Syn/CYS/FTO, and α-Syn/anti-α-Syn/CYS/FTO electrodes, respectively. Inset of (**a**) shows the *I_pa_* and *ΔE_pp_* and the upper inset of (**b**) displays the *R_ct_* for the preparation of CYS/FTO electrodes with varying incubation time, respectively. The lower inset of (**b**) displays Randles equivalent circuit model to fit the Nyquist plots, where, *R_s_* is solution resistance, *Z_w_* is the Warburg diffusion resistance, and *C_dl_* is the double-layer capacitance. The circles and solid line in (**b**) specifies the experimental and fitted EIS data, respectively.

Figure 3a displays the cyclic voltammograms (CVs) of the bare FTO and CYS/FTO electrodes in a mixture solution of [Fe(CN)_6_]^3−/4−^ redox couple in PB (pH 7.0), while Figure 3b shows the corresponding Nyquist plots. The anodic peak current (*I_pa_*) and peak-to-peak separation (*ΔE_pp_*) for the redox reaction of [Fe(CN)_6_]^3−/4−^ were ca. 0.41 mA and ca. 0.29 V, respectively. Upon the formation of CYS-SAM onto the FTO electrode, the redox activity and the reversibility of the [Fe(CN)_6_]^3−/4−^ was decreased significantly with the *I_pa_* and *ΔE_pp_* of ca. 0.24 mA and 0.53 V, respectively. The decrease of the redox behavior at the CYS/FTO electrode can be assigned to the higher electrostatic perturbation between the negatively charged redox couple with the enhanced negatively charged electron density at the CYS/FTO surface induced by the tail –NH_2_ functional groups of CYS-SAM [33]. While the insulating nature of CYS is also partly responsible for the reduced redox activity and catalytic activity for [Fe(CN)_6_]^3−/4−^. The redox behavior of the [Fe(CN)_6_]^3−/4−^ at the bare FTO and CYS/FTO electrode is highly consistent with the variation of *R_ct_*, which was ca. 2.79 and 3.85 kΩ, respectively, for FTO and CYS/FTO, respectively. Before the application of the CYS/FTO electrode for the immobilization of anti-α-Syn antibody and detection of α-Syn antigen, we optimized the incubation time for the preparation of CYS/FTO electrode by CV and EIS analyses. Insets of Figure 3a,b show the summary of the CV and EIS parameters (*I_pa_*, *ΔE_pp_*, and *R_ct_*), respectively, for the preparation of the CYS/FTO electrode with different incubation time. The *I_pa_* for the [Fe(CN)_6_]^3−/4−^ redox mediator was decreased continuously, while both *ΔE_pp_* and *R_ct_* were increased with the increase of the incubation time from 1 to 8 h. After 4 h, all these parameters were almost unchanged. This designates that the surface coverage of FTO reached almost saturation after 4 h due to SAM formation. Thus, 4 h was regarded as the optimal incubation time for the preparation of SAM for the following experiments.

The immobilization of anti-α-Syn and the binding of α-Syn was studied by CV and EIS analyses, as shown in Figure 3a,b, respectively. The *I_pa_* of the anti-α-Syn/CYS/FTO electrode was ca. 0.21 mA, which was 13.5% lower than the *I_pa_* of CYS/FTO electrode. This decreased redox activity of the anti-α-Syn/CYS/FTO electrode compared to the CYS/FTO electrode for [Fe(CN)_6_]^3−/4−^ demonstrate the effective immobilization of anti-α-Syn by the formation of peptide bond between the –NH_2_ functional groups of CYS and –COOH groups of anti-α-Syn [22,23]. The decreased *I_pa_* at the anti-α-Syn/CYS/FTO can be attributed to the insulating character of the anti-α-Syn protein, which acts as a barrier for interfacial electron transfer [22,23]. Accordingly, the *ΔE_pp_* and *R_ct_* were increased at the anti-α-Syn/CYS/FTO, which were ca. 0.58 V and 4.32 kΩ, respectively. Upon the binding of α-Syn (50 ng/mL) onto the anti-α-Syn/CYS/FTO electrode, the *I_pa_* and *ΔE_pp_* were ca. 0.16 mA and 0.64 V, respectively. The additional decrease of the *I_pa_* and the widening of *ΔE_pp_* imply the further reduction of the electrocatalytic ability of the redox reaction of [Fe(CN)_6_]^3−/4−^. This specifies the successful immunocomplex formation between the anti-α-Syn antibody and α-Syn antigen [15,23]. The successful binding of α-Syn antigen can be further verified by the additional increment of *R_ct_* (ca. 5.32 kΩ) at the α-Syn/anti-α-Syn/CYS/FTO electrode, which was ca. 23% higher compared to the anti-α-Syn/CYS/FTO electrode.

### 3.3. Detection of α-Syn Antigen and Selectivity Study

**Figure 4:**
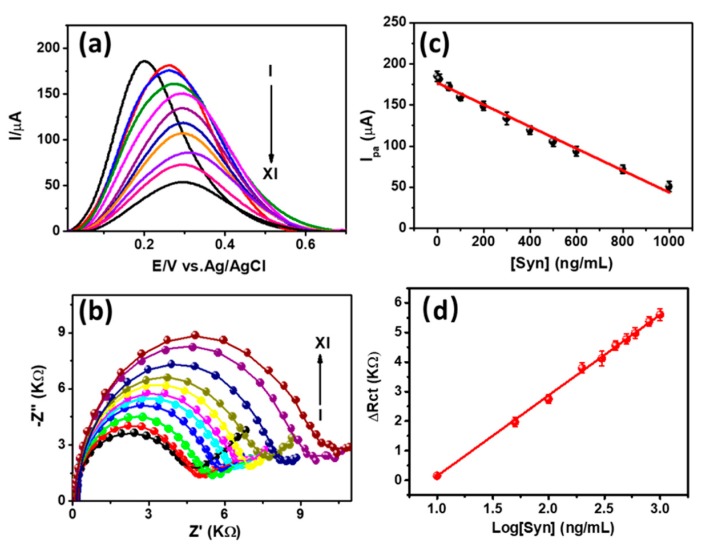
(**a**) Differential pulse voltammetry (DPV) responses and (**b**) EIS spectra for [Fe(CN)_6_]^3−/4−^ after the binding of α-Syn at the immunosensor probe with different concentrations (I→XI: 0, 10, 50, 100, 200, 300, 400, 500, 600, 800, and 1000 ng/mL); (**c**,**d**) are the corresponding calibration plots obtained from the DPV and EIS responses, respectively, with the standard error value of 5%. All the DPV signals are presented after baseline correction. The circles and solid line in Figure 4b designate the experimental and fitted EIS data, respectively.

Figure 4a displays the DPV response for the oxidation of [Fe(CN)_6_]^3−/4−^ after the attachment of α-Syn antigen at the anti-α-Syn/CYS/FTO immunosensor probe with different concentrations (10–1000 ng/mL). Similar registered currents with low standard deviation were obtained due to the fixed experimental conditions such as the geometric area and the concertation Fe(CN)_6_]^3−/4−^ redox couple. The *I_pa_* of the DPV responses were decreased with the increase of the [α-Syn]. Additionally, the oxidation peak potential (*E_pa_*) was continuously shifted to positively with the increase of the [α-Syn]. This can be ascribed to the increase of the charge perturbation for the oxidation of negatively charged [Fe(CN)_6_]^3−/4−^ induced by the negatively charged α-Syn [34]. The *I_pa_* showed a linear behavior against [α-Syn] with the linear regression equation of *I_pa_* (mA) = −0.133 ± 0.004 × [α-Syn] (ng/mL) + 176.88 ± 2.15, (R^2^ = 0.988), as shown in Figure 4c. This resulted in the detection limit and sensitivity of ca. 3.62 ng/mL and ca. 133 µA/ng/mL, respectively, with the signal-to-noise (*S*/*N*) of 3. Further, we detect the [α-Syn] by the EIS method since it exhibits better analytical performance. Figure 4b depicts the Nyquist plots for the binding of α-Syn antigen at the immunosensor probe with the concentration ranging from 10 to 1000 ng/mL. The diameter of the semicircle was increased with increasing the [α-Syn], which is consistent with the DPV responses. The variation of difference in *R_ct_* showed a linear behavior with the logarithmic concentration of α-Syn, as shown in Figure 4d. This corresponds to the linear regression equation of *ΔR_ct_* (kΩ) = 2.72 ± 0.019 × Log[α-Syn] (ng/mL) − 2.57 ± 0.028, (R^2^ = 0.998), which resulted the detection limit of ca. 1.13 ng/mL (*S*/*N* = *3*). The analytical performance of the present immunosensor is comparable with the reported other α-Syn immunosensors and aptasensors, as summarized in Table 1.

**Figure 5:**
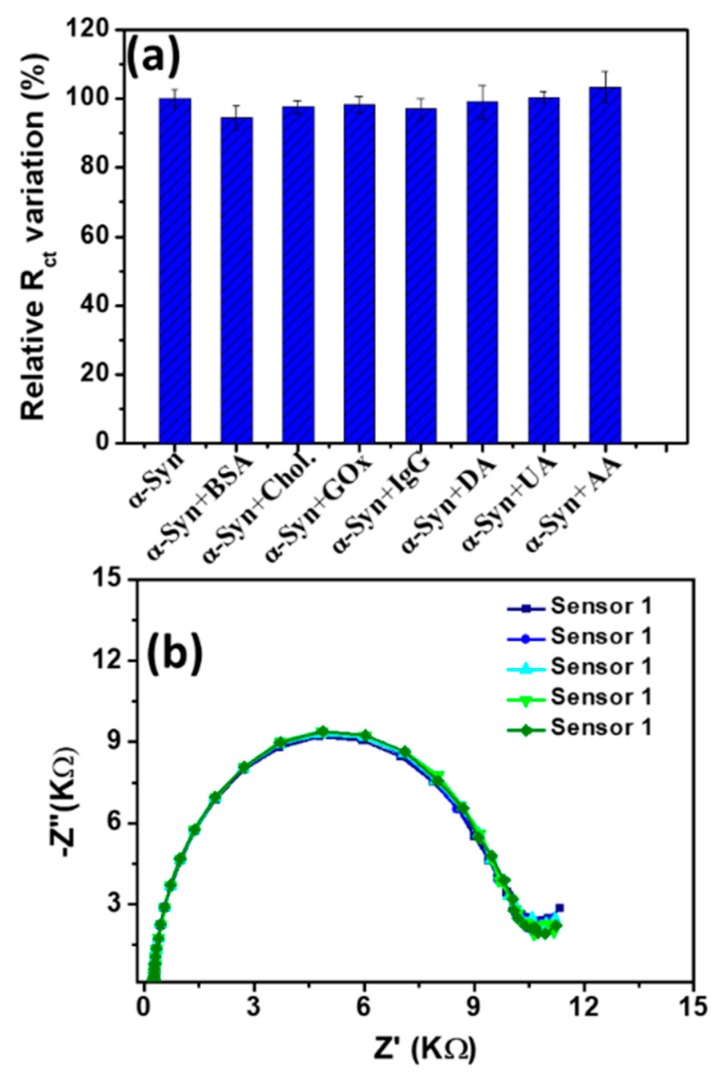
(**a**) Relative variation of the *R_ct_* obtained from EIS measurements in the absence and presence of different interferences. The relative variation of the *R_ct_* was measured three times in the presence of each interference, which was averaged and shown in (**a**) with the standard error value of 5%. (**b**) Nyquist plots of five independent immunosensor after the attachment of α-Syn (1000 ng/mL).

The selectivity of the immunosensor was studied using some common interfering proteins and compounds which usually co-exist in biological fluids with α-Syn, including Chol., BSA, GOX, IgG, DA, AA, and UA. The concentration of these interferences was five-fold higher compared to the target α-Syn (200 ng/mL). Figure 5a displays the relative variation of *R_ct_* [Fe(CN)_6_]^3−/4−^ after the attachment of α-Syn at the immunosensor probe in the presence and absence of interferences. The variation of the *R_ct_* was less than ca. 10%, demonstrating the high selectivity and specificity of the proposed immunosensor. 

### 3.4. Reproducibility, Stability, and Real Sample Analysis

The reproducibility of the sensor was investigated by the EIS measurements of five individual α-Syn (1000 ng/mL) attached immunosensors. The corresponding Nyquist plots are shown in Figure 5b, which showed a low relative standard deviation (RSD) of the *R_ct_* (ca. 1.3%). This designates the high reproducibility of the immunosensor. The electrochemical stability of the immunosensor was tested by the consecutive EIS measurements (10 times) after the binding of α-Syn (1000 ng/mL) at the immunosensor probe. The variation of the *R_ct_* was significantly low with the RSD of only ca. 2.50%. Additionally, the sensor retained ca. 93.68% of its initial *R_ct_* value after being stored at 4 °C for 7 days in PB solution, designating the high storage stability of this immunosensor. The good stability could attribute to the strong SAM formation by adsorption onto FTO electrode, the stable binding of antibody and antigen, the proteins adsorption as well which need to be investigated more intensively in future.

The practical applicability of the present immunosensor was examined using the different concentrations of α-Syn (50, 200, and 500 ng/mL), which was spiked in the diluted human serum (100 times with PB) samples. The recoveries of the sensor were measured by calculating the *R_ct_* from the EIS plots, which were in the range from ca. 95.8–101.3% (Table 2). This designates that the immunosensor is promising for the detection of α-Syn in blood plasma with high accuracy. 

## 4. Conclusions

In summary, we have successfully fabricated a simple, low-cost, sensitive, and selective electrochemical immunosensor to detect α-Syn, which is a potential biomarker of PD. The anti-α-Syn antibody was covalently attached to a CYS-SAM modified FTO electrode, which enables the formation of immunocomplex between the anti-α-Syn antibody and α-Syn antigen. This induces the variation of the redox behavior of the [Fe(CN)_6_]^3−/4−^ redox couple at different stages of modifications. Upon the binding of α-Syn antigen onto the anti-α-Syn/CYS/FTO immunosensor probe, the oxidation peak current of the redox couple was decreased with the increase of the *R_ct_*. This resulted in a wide linear range for α-Syn detection from 10 to 1000 ng/mL with a low detection limit (*S*/*N* = *3*) of ca. 3.62 and 1.13 ng/mL obtained from DPV and EIS measurement, respectively. The immunosensor exhibited good interference-resistance, stability, reproducibility, and good recoveries of α-Syn detection in the human serum samples.

## Figures and Tables

**Table 1:** Comparison of the analytical performances of different reported α-Syn immunosensors and aptasensors with the present immunosensor.

Electrode Configuration	Sensor Type	Measurement Method	Linear Range (ng/mL)	LOD (ng/mL)	Ref.
Au–TiO_2_ NTs	Immunosensor	Photo-electrochemical	0.05–100	0.034	[18]
Thiolated Au	Aptasensor	EIS, SPR	0.1 nM–0.5 μM	0.001	[19]
Apt-CS-Au	Aptasensor	CV, DPV	60 pM–150 nM	10 pM	[20]
Au NP–PGA /ITO	Immunosensor	EIS, CV, SWV	0.004–2	0.135	[22]
PAMAM–Au/C	Immunosensor	EIS, CV	0.02–200	0.0146	[23]
CYS/FTO	Immunosensor	DPV, EIS	10–1000	3.62, 1.13	This work

Notes: NTs: nanotubes; PAMAM: amine-terminated polyamidoamine; SWV: square wave voltammetry; SPR: surface Plasmon resonance; HMDE: hanging mercury drop electrode; PGA: poly-glutamic acid.

**Table 2:** Recovery results for the detection of α-Syn in diluted human serum samples.

Sample No.	[α-Syn] Added (ng/mL)	[α-Syn] Found (ng/mL) ^a^	Recovery (%)	RSD (%)
1	50	47.9 ± 2.7	95.8 ± 5.4	1.87
2	200	198.4 ± 5.2	99.2 ± 2.6	0.98
3	500	506.5 ± 9.6	101.3 ± 1.9	3.24

^a^ average of three measurements.
